# Subsidence of the Corail stem in total hip arthroplasty: no influence of bony contact

**DOI:** 10.1186/s10195-024-00794-y

**Published:** 2024-11-10

**Authors:** Filippo Migliorini, Nicola Maffulli, Marco Pilone, Daniel Kämmer, Ulf Krister Hofmann, Andrea Nobili, Erlis Velaj, Andreas Bell

**Affiliations:** 1 Department of Orthopaedic, Trauma, and Reconstructive Surgery, University Hospital Aachen, RWTH Aachen University Hospital, Pauwelsstraße 30, 52074 Aachen, Germany; 2 Department of Orthopaedic and Trauma Surgery, Academic Hospital of Bolzano, 39100 Bolzano, Italy; 3 Department of Life Sciences, Health, and Health Professions, Link Campus University, 00165 Rome, Italy; 4 Department of Trauma and Orthopaedic Surgery, Faculty of Medicine and Psychology, University “La Sapienza” of Rome, Rome, Italy; 5 School of Pharmacy and Bioengineering, Keele University School of Medicine, Thornburrow Drive, Stoke on Trent, England; 6 Barts and the London School of Medicine and Dentistry, Centre for Sports and Exercise Medicine, Queen Mary University of London, Mile End Hospital, 275 Bancroft Road, London, E1 4DG England; 7 Residency Program in Orthopedics and Traumatology, University of Milan, Milan, Italy; 8 Department of Orthopaedic and Trauma Surgery, Eifelklinik St. Brigida, 52152 Simmerath, Germany

**Keywords:** Hip, Arthroplasty, Stem subsidence, Implant bone, Contact

## Abstract

**Introduction:**

This study investigated stem subsidence following primary total hip arthroplasty (THA) with a Corail stem in patients who underwent two-staged bilateral THA. The second outcome of interest was to investigate whether a specific single cortical bone contact point might reduce postoperative stem subsidence.

**Methods:**

The present study was conducted following the STROBE guidelines. The records of patients who underwent THA between 2016 and 2023 were accessed. All patients who underwent two-staged bilateral THA were retrieved. The direct contact between the stem and the cortical bone was assessed at various points in the metaphysis and the distal portion of the stem (diaphysis) in both anteroposterior radiographs of the pelvis (medial and lateral bone contact) and a Lauenstein view of the hip (anterior and posterior bone contact). The following parameters were measured and compared to assess stem subsidence: distance from the proximal femur at the stem bone interface and the tip of the lesser trochanter (distance A); distance from the tip of the lesser trochanter and the tip of the femoral stem (distance B).

**Results:**

In total, 250 patients were included, 45% (149 of 250 patients) were women and 61% (153 of 250 THAs) were implanted primarily on the right side. The mean age of patients at the time of the first THA was 64.3 ± 10.0 years and the mean body mass index (BMI) was 28.0 ± 4.9 kg/m^2^. The mean length of the follow-up was 14.1 ± 10.8 months. The overall stem subsidence following THA was 2.8 ± 0.7 mm (*P* < 0.006). A direct cortical bone-implant contact did not exert a statistically significant difference in subsidence of the THA stem at the metaphysis and diaphysis (*P* > 0.5). Stem subsidence following THA with a collarless cementless Corail stem was approximately 2.8 mm at 14 months.

**Conclusions:**

Direct cortical bone contact of the stem at diaphysis and metaphysis seems not to influence stem subsidence following THA using the Corail stem.

## Introduction

Total hip arthroplasty (THA), performed in patients with end-stage joint osteoarthritis, restores joint function, increasing patients’ quality of life and mobility [1–3]. In cementless THAs, the femoral component integrates with the surrounding bone [4–7]. This process, namely osteointegration, is crucial to ensure long-term implant survival [4, 8]. However, the femoral component of THA, although well integrated with the surrounding bone, can undergo subsidence over time [9, 10]. Stem subsidence greater than 5 mm can lead to implant failure [11]. Indeed, aseptic loosening of the implant is a major cause of revision THA [12–17]. In current literature, evidence on stem subsidence is scarce, and the underlying causes of stem subsidence have not yet been fully clarified. Several factors could promote stem subsidence, such as stem design, patient characteristics, bone turnover and remodelling [18–20].

The effect of cortical bone contact on stem subsidence in THA has been poorly investigated. The primary outcome of interest in the present study was to assess the amount of stem subsidence following primary THA using a cementless collarless Corail stem. Subsidence was assessed using the anteroposterior pelvis radiographs of patients who underwent two-staged bilateral THA. The anteroposterior radiographs taken after the first THA were compared with those of the same side taken at the time of the contralateral THA.

## Methods

### Study design

The present study was performed using the Strengthening the Reporting of Observational Studies in Epidemiology (STROBE) [21] as a retrospective analysis of prospectively collected data. The databases of the Department of Orthopaedic Surgery of the Eifelklinik St. Brigida, Simmerath, Germany and the Department of Orthopaedic, Trauma, and Reconstructive Surgery of the University Hospital RWTH Aachen, Germany, were accessed. The records of all patients who had undergone THA between 2016 and 2023 were accessed for inclusion. Informed consent was obtained from every patient to use medical data for research purposes. The present study was approved by the ethics committee of the RWTH University of Aachen (project ID: EK128/19) and conducted according to the principles expressed in the Declaration of Helsinki.

### Eligibility criteria

Inclusion criteria were: (1) symptomatic idiopathic hip osteoarthritis (OA) or OA secondary to dysplasia or femoral head necrosis, (2) symptomatic OA grade II–IV according to the Kellgren–Lawrence classification [22], (3) completion of postoperative antithrombotic prophylaxis, (4) completion of postoperative prophylaxis for heterotopic ossification, (5) a minimum of 6 months between the implantation of the ipsi- and the contralateral THA and (6) patients being able to understand the nature of treatment. Exclusion criteria were: (1) hip OA secondary to trauma; (2) neoplastic diseases; (3) pregnancy; (4) any blood abnormalities; (5) severe peripheral neuropathy, vascular diseases or presence of peripheral ulcers; (6) implantation of a cemented stem; (7) simultaneous intake of anticoagulants or calcitonin; (8) patients who had undergone revision surgery for any reason; and (9) other omitted criteria which may have influenced the results of the present investigation.

### Surgical technique

All patients received a 1.5 g single shot of intravenous cefuroxime 20 min before induction of anaesthesia. Six senior surgeons performed all surgeries using the Watson–Jones anterolateral approach [23]. The implant used for THA was the cementless DePuy (DePuy Synthes, Raynham, MA, USA) Corail stem and a Pinnacle acetabular cup, an oxinium or ceramic femoral head, and a high-molecular weight crosslinked polyethylene (XLPE) liner. Anti-thrombotic prophylaxis with 10 mg of rivaroxaban daily for 6 weeks, started 12 h after the index procedure was implemented. Prophylaxis for heterotopic ossification was performed with 600 mg of ibuprofen thrice daily for 6 weeks. A team of physiotherapists followed patients during hospitalisation. Quadriceps strength exercise started on the first postoperative day, and the patient mobilised weight bearing as tolerated using a forearm support frame. By the third postoperative day, patients usually progressed to mobilisation using crutches. An outpatient rehabilitation program was set up and personalised for every patient.

### Outcomes of interest

**Fig. 1:**
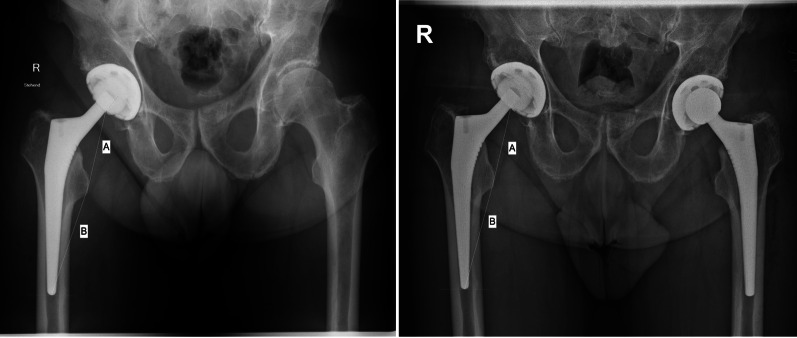
Evidence of subsidence of 1.3 mm after 4 years follow-up in a 68 year old female patient on anteroposterior pelvis radiographs (**A** **left**: following the first THA; **B** **right**: following the implantation of the contralateral THA)

The present investigation considered all patients who had undergone two-staged bilateral THA. Data concerning the date of surgery, age, sex, weight and height of the patients were collected. On admission, patients received an anteroposterior radiograph of the pelvis and a Lauenstein view of the hip. The anteroposterior radiographs taken following the first THA were compared with those of the same side taken at the time of the contralateral THA (Fig. 1).

**Fig. 2:**
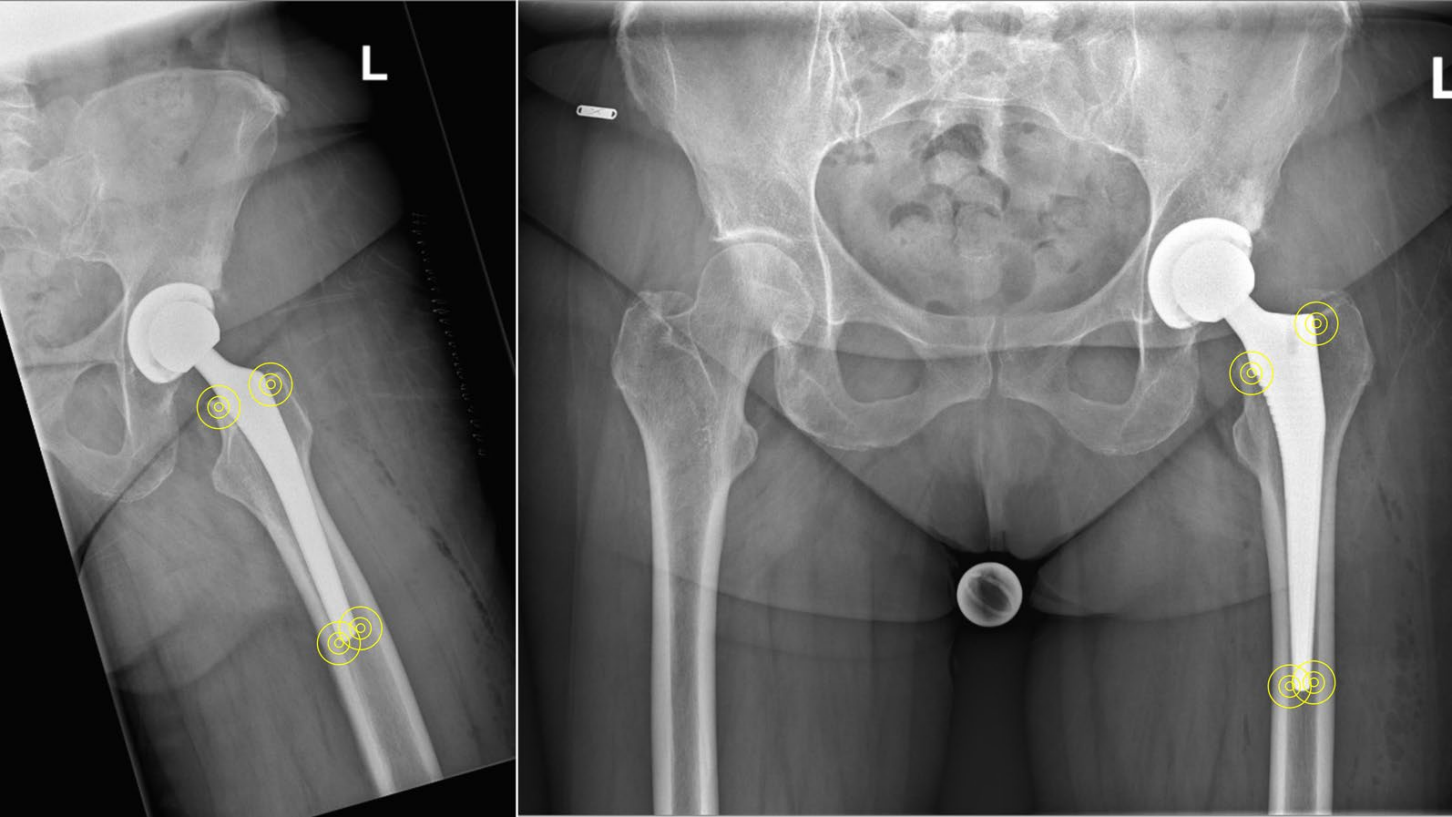
Assessment of direct cortical bone contact of the implanted stem

The contact between cortical bone and implant stem was assessed at the metaphysis and the distal portion of the stem in both the anteroposterior pelvis radiographs and a Lauenstein view of the hip. The contact was evaluated at each side (lateral, medial, anterior and posterior) and at the proximal (metaphysis) and distal (diaphysis) implant regions (Fig. 2). Two experienced surgeons (A.B. and A.N.) evaluated the presence of bone-implant contact on all radiographs separately. The bone contact was evaluated as a dichotomous trait: ‘direct-contact’ or ‘no-direct-contact’. These surgeons work in a high-volume centre that performs approximately 1000 THA annually and is certified EndoCert (Centres of German Endoprosthetic, German Society for Orthopedics and Traumatology). The EndoCert initiative represents the first worldwide certification system of medical centres for total joint replacement and was established in Germany in 2012. The EndoCert aims to maintain quality standards in primary and revision arthroplasty. The associated centres also develop and define standards and treatment processes, and they are subject to continuous re-certification [29, 30]. Only radiographs with 100% concordance (YY or NN) were included in the present study. Direct cortical bone contact of the stem was evaluated on the first postoperative radiographs of the affected hip after implantation of the first THA. The degree of subsidence was evaluated by comparing the ipsilateral anteroposterior radiographs of the pelvis conducted at the time of the first THA with those obtained at the time of the implantation of the contralateral THA. By doing so, patients were not exposed to additional radiographs for research purposes.

**Fig. 3:**
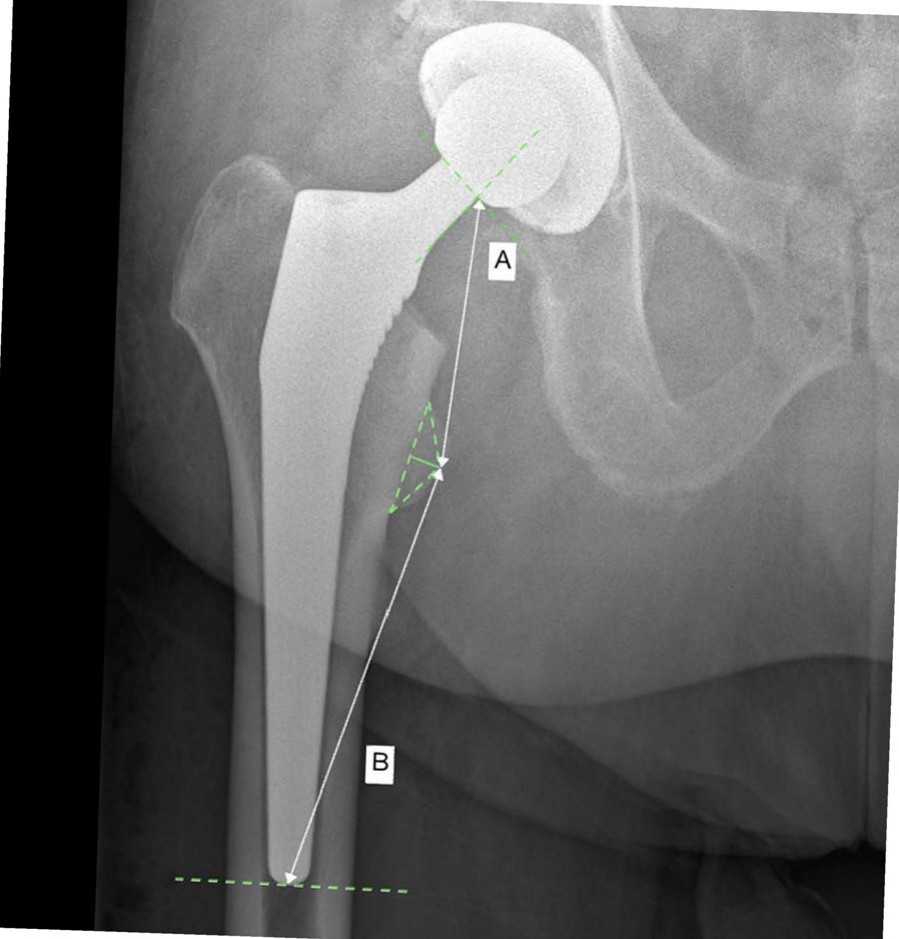
Reference parameters to measure stem subsidence (**A** **left**: distance from the proximal femur at the stem bone interface and the tip of the lesser trochanter; **B** **right**: distance from the tip of the lesser trochanter and the tip of the femoral stem). Stem subsidence was then calculated as the mean of absolute differences for the distances A and B between the first and second x-rays

The amount of subsidence was assessed by a blinded assessor who was not involved in the clinical management of the patients. The imaging references used to assess stem subsidence are shown in Fig. 3. In the present investigation, the radiographs were divided into two groups: imaging showing evidence of cortical bone-implant contact and those showing no cortical bone-implant contact. For each group, the amount of subsidence (length of distances A and B) was evaluated at each side (lateral, medial, anterior, and posterior) of the proximal (metaphysis) and distal (diaphysis) implant regions.

### Statistical analysis

The main author (**) performed the statistical analyses using the IBM SPSS software (version 25). For descriptive statistics, arithmetic mean and standard deviation were evaluated. The mean difference (MD) effect measure was calculated for continuous variables. The two-tailed paired T-test was performed to evaluate the overall amount of stem subsidence. The analyses were conducted separately for each stem portion (metaphysis or diaphysis), and the amount of subsidence (length of distances A and B) was compared between the bony contact and non-contact groups at the lateral, medial, anterior and posterior bony-implant interface. The two-tailed unpaired *t*-test was performed to evaluate possible differences between the contact and non-contact groups. Values of *P* < 0.05 were considered statistically significant.

## Results

### Recruitment process

**Fig. 4:**
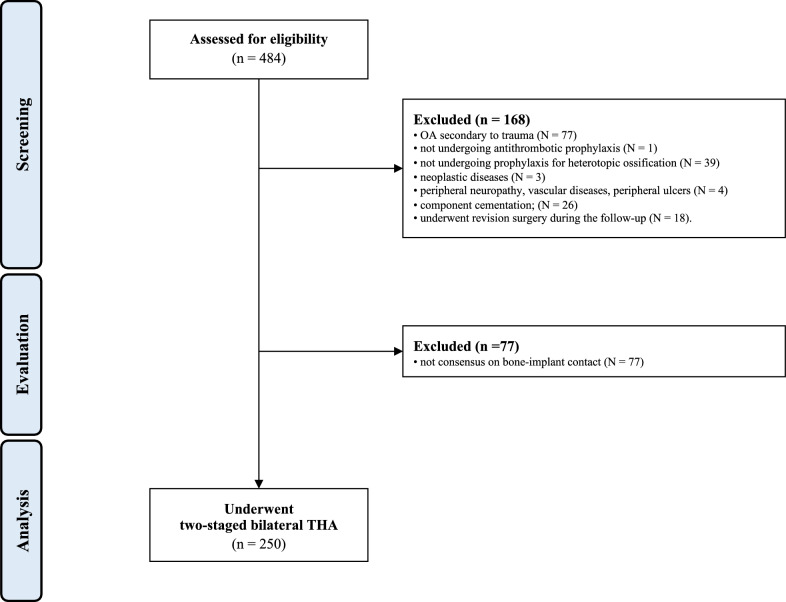
Diagram of the recruitment process

Data from 484 procedures were retrieved. Of them, 168 were not considered in the present study for the following reasons: OA secondary to trauma (*N* = 77); not undergoing antithrombotic prophylaxis (*N* = 1); not undergoing prophylaxis for heterotopic ossification (*N* = 39); neoplastic diseases (*N* = 3); severe peripheral neuropathy, vascular diseases or presence of peripheral ulcers (*N* = 4); component cementation (*N* = 26); or underwent revision surgery during the follow-up (*N* = 18). Additionally, 77 radiographs were excluded as they did not report between-authors consensus on the presence of direct bony-implant contact. Finally, 250 patients were identified and included in the present analysis (Fig. 4).

### Patient demographics

Overall, 250 patients were included, 45% (149 of 250 patients) were women and 61% (153 of 250 THAs) were implanted primarily on the right side. The mean age of patients at the time of the first THA was 64.3 ± 10.0 years, and the mean body mass index (BMI) was 28.0 ± 4.9 kg/m^2^. The mean length of the follow-up was 14.1 ± 10.8 months (range, 6—23). Demographic data are shown in Table 1.

**Table 1:** Demographic data of the patients (FU: follow-up)

Endpoint	Value
Side (right)	61% (153 of 250)
Women	45% (149 of 250)
BMI (kg/m^2^)	28.0 ± 4.9
Age (years)	64.3 ± 10.0
FU (months)	14.1 ± 10.8

### Outcomes of interest

The overall stem subsidence following THA was 2.8 ± 0.7 mm (*P* < 0.006). Table 2 reports the amount of subsidence in zones A and B.

**Table 2:** Main results

Endpoint	At baseline	At last FU	MD	T	95% CI	*P*
A	58.1 ± 7.3 (75.7–29.2)	55.8 ± 7.9 (73.1–28.3)	2.3	3.381	3.6366 to 0.9634	0.0008
B	106.8 ± 15.0 (134.2–57.9)	110.1 ± 14.8 (138.5–59.9)	3.3	2.476	0.6815 to 5.9185	0.01

### Effect of metaphyseal cortical bone contact

A metaphyseal direct cortical bone implant did not influence the subsidence of the THA stem at each evaluated point (Table 3).

**Table 3:** Results of metaphyseal single cortical bone contact points

Metaphysis	No contact	Yes contact	No contact	Yes contact	MD	Effect size	*T*	*P*
A lateral	2	248	–	–	–	–	–	–
B lateral	2	248	–	–	–	–	–	–
A medial	114	136	1.9 ± 3.6	2.2 ± 2.9	0.3	0.083	−0.697	0.5
B medial	114	136	2.6 ± 5.2	3.5 ± 4.2	0.9	0.19	−1593	0.1
A anterior	11	239	2.9 ± 3.2	2.0 ± 3.2	0.9	0.58	2.26	0.05
B anterior	11	239	2.1 ± 7.8	3.3 ± 4.4	1.4	0.73	−1.714	0.1
A posterior	84	166	1.8 ± 3.2	3.2 ± 3.3	1.4	0.12	−0.9711	0.3
B posterior	84	166	2.4 ± 4.7	3.5 ± 4.7	1.9	0.23	−1.8854	0.06

### Effect of diaphyseal cortical bone contact

A metaphyseal direct cortical bone implant did not influence the subsidence of the THA stem at each evaluated point (Table 4).

**Table 4:** Results of diaphyseal single cortical bone contact points

Diaphysis	No contact	Yes contact	No contact	Yes contact	MD	Effect size	*T*	*P*
A lateral	139	111	2.0 ± 3.8	2.2 ± 2.4	0.2	0.083	−0.7325	0.5
B lateral	139	111	3.2 ± 5.3	2.9 ± 3.9	0.3	0.075	0.6591	0.5
A medial	156	94	1.8 ± 3.0	2.4 ± 3.5	0.6	0.17	−1.434	0.2
B medial	156	94	2.8 ± 4.3	3.4 ± 5.2	0.6	0.11	−0.9438	0.4
A anterior	239	11	3.0 ± 3.2	3.7 ± 2.9	0.7	0.52	−2.3479	0.06
B anterior	239	11	3.1 ± 4.9	3.4 ± 1.5	0.3	0.068	−0.7062	0.5
A posterior	36	214	3.2 ± 3.7	2.9 ± 3.1	0.3	0.42	2.2829	0.09
B posterior	36	214	3.0 ± 6.1	3.3 ± 4.4	0.3	0.28	−1.3422	0.2

## Discussion

According to the main findings of the present study, stem subsidence following THA was approximately 2.8 mm at an average of 14 months from implantation. Of note, this amount of subsidence equals approximately the difference in leg length achieved by changing the head size from, for example, M to L in a stem with a CCD-angle of 135° (approximately 2.8 mm). At lower CCD-angles, the effect of the subsidence would be more pronounced. When implanting a Corail stem without a collar, no single direct bone contact with the stem at the diaphysis or metaphysis affected the degree of stem subsidence following THA.

A Corail stem without a collar was implanted in all patients in our study. The Corail stem is a straight implant with a quadrangular cross-section. Compaction broaching is the ideal implantation technique recommended by the manufacturer, allowing the preservation of bone stock and maintaining periosteal blood supply [24]. Khanuja et al. [25] classified straight cementless stems into six categories according to the shape of the implant and its surface. The initial stability of the implant and the consequent cortical contact depend on stem geometry [26]. The Corail stem is classified as type 3 C, and it is characterised by diaphyseal and metaphyseal fixation [25].

Choosing the proper implant size is fundamental to avoid periprosthetic fracture and implant mobilisation and to minimise surgical time [27, 28]. Kobayashi et al. [29] classified the Corail stem (DePuy, Leeds, UK) cortical contact into five categories using computed tomography (CT) scans of 55 patients (Table 5).

**Table 5:** Results of diaphyseal cortical bone contact Kobayashi classification [29]

Type	Description	Cortical contact (%)
1	Neither proximal nor distal cortical contact	3
2	Proximal cortical contact	12
3	Distal cortical contact	10
4	One proximal and one distal cortical contact area	30
5	At least one cortical contact area and more than one distal cortical contact area	45

Interestingly, after 31 months, no statistically significant difference in clinical and radiographic scores was shown among these five groups and no revision surgery was performed. Sanki et al. [30] classified the stem cortical contact in a collared fit-and-fill stem design according to the number of coronal and sagittal plane contact areas. High contact (≥ 7 contact areas), medium contact (≥ 4 and ≤ 7 contact areas) and low contact (≤ 4 contact areas), using CT images to evaluate the stem position in 100 hips. Interestingly, severe stress shielding was observed in the high-contact group. In contrast, no severe stress shielding was seen among the low-contact group. Stress shielding can produce periprosthetic bone reabsorption, leading to aseptic loosening [31], a risk factor for periprosthetic fractures [32]. For these reasons, the authors suggest undersizing the stem [30]. Undersizing of the stem may, however, also lead to implant failure [28, 33]. The technical manual of the operative technique of the Corail stem suggests that there should be a 1 mm margin of compacted cancellous bone around the stem [34]. The correct stem size can be established using the Canal Fill Index (CFI) [35]. The stem is considered undersized if the CFI is ≤ 80% [36]. McConnel et al. [37] retrospectively analysed 1337 Corail stems and proposed a system to classify the undersized Corail stem into four categories: uniformly undersized, varus undersized, valgus undersized and cocktail-glass undersized. This classification is not based on the bone-implant contact ratio but on the spatial relationship between the stem and the cortical bone. The present study evidenced no association between the diaphysis nor the metaphysis cortical contact and subsidence when considering single contact points. McConnel et al. [37] also proposed a radiographic classification without relating imaging assessment to the clinical outcome.

It is crucial to underline the difference between the association between the under sizing of the implant and subsidence, discussed in the preceding paragraph, and the association between the size of the implant and subsidence. Leiss et al. [38] conducted a study on 114 patients with a collarless uncemented stem, dividing the cohort into two groups: early full and partial weight-bearing. No association between stem size and subsidence was found in other groups. There are still controversial results regarding the influence of cortical contact on subsidence [30, 39, 40]. Sanki et al., for example, did not find any difference in subsidence among the three groups [30]. Inoue et al. [39], in contrast, analysed stem subsidence in 75 hips and observed an association between stem subsidence and the lack of cortical contact at the distal portion of the implant. Reimeringer et al. [41] interestingly described an increment in stem micromotion and a decrement in primary stability when distal cortical contact was present. These discordances may depend on the implant geometry [25], supported by the congruence of the present study and Sanki’s results, both based on the Corail stem.

The present study showed a mean subsidence of 2.8 mm at 14 months. Ries et al. [42] analysed the subsidence of 231 Corail stems after 7 months, and a mean subsidence of 2.9 mm was found. Of note, the subsidence rate was statistically significantly higher in collarless femoral stems (3.1 mm) versus collared stems (1.9 mm). Subsidence occurs mostly in the first 3 months from implantation and tends to stabilise before 24 months [43, 44]. In our department, in patients who require bilateral THA, we recommend undergoing surgery on the contralateral side for at least 6 months following the index procedure. Therefore, the most clinically relevant subsidence already occurred. Data of patients with longer follow-up than 24 months was limited; therefore, we included patients with a minimum of 6 months follow-up to increase data pooling. Osseointegration starts to be radiographically evident after 3 months [45]. Primary stabilisation from implant positioning influences the stability of the stem before the ossification process takes place [46]. Park et al. [47] showed that when a 40% contact ratio is achieved, a further increment in contact ratio does not influence implant micromotion and primary stability.

We are aware that this methodology has limitations. However, we point out that it is of simple execution and quick application. Only radiographs with 100% concordance (YY or NN) were included in the present study. No additional assessment was performed in disagreements, and the patient was excluded for analysis. This modality might enhance the risk of selection bias and reduce the reliability of the results of the present study. Still, it also allowed us to study patients in whom the assessors had achieved immediate full concordance. The modality of subsidence evaluation might be influenced by sinusoidal function during flexion, which might have influenced the reliability of the results of the present study. The prevention of thromboembolism and heterotopic ossification is debated [3, 48–51]. We also excluded patients who had not undergone antithrombotic prophylaxis and those who did not undergo prophylaxis for heterotopic ossification to reduce variability in the postoperative protocol; however, whether the prevention of thromboembolism and/ or heterotopic ossification influences stem subsidence is unclear.

## Conclusion

Our analysis shows that stem subsidence following THA with a collarless cementless Corail stem was approximately 2.8 mm at a mean of 14 months of follow-up. Direct cortical bone contact of the stem at diaphysis and metaphysis might not influence stem subsidence following THA using this implant.

## Data Availability

The data underlying this article are available at reasonable request to the main author FM (Migliorini.md@gmail.com).
